# Advanced Echocardiography Assessment in the Management of Alcapa Syndrome: Case Report

**DOI:** 10.3390/jcdd11070219

**Published:** 2024-07-11

**Authors:** Asmaa Carla Hagău, Horațiu Suciu, Anca Voichița Popoiu, Iolanda Muntean

**Affiliations:** 1 Doctoral School of Medicine and Pharmacy, I.O.S.U.D., George Emil Palade University of Medicine, Pharmacy, Science, and Technology of Târgu Mureș, 540136 Târgu Mureș, Romania; 2 Clinic of Paediatric Cardiology, Emergency Institute for Cardiovascular Diseases and Transplantation of Târgu Mureș, 540139 Târgu Mureș, Romania; iolanda.muntean@umfst.ro; 3 Department of Surgery IV, Emergency Institute of Cardiovascular Diseases and Transplantation Târgu Mureș, 540139 Târgu Mureș, Romania; horatiu.suciu@umfst.ro; 4 Department of Surgery, George Emil Palade University of Medicine, Pharmacy, Science, and Technology of Târgu Mureș, 540142 Târgu Mureș, Romania; 5 Department of Pediatrics, Children’s Hospital “Louis Turcanu”, 300732 Timisoara, Romania; apopoiu@umft.ro; 6 Department of Pediatrics, University of Medicine and Pharmacy “Victor Babes” Timisoara, 300732 Timisoara, Romania; 7 Department of Pediatrics III, George Emil Palade University of Medicine, Pharmacy, Science, and Technology of Târgu Mureș, 540142 Târgu Mureș, Romania

**Keywords:** alcapa, echocardiography, speckle tracking, acute heart failure, congenital heart defects

## Abstract

Anomalous origin of the left coronary artery from the pulmonary artery (ALCAPA) is a rare and potentially life-threatening condition affecting infants that requires immediate corrective surgery to restore blood flow to the myocardium. We present a case of an infant with ALCAPA and severe heart failure. What sets this case apart is the utilization of speckle-tracking echocardiography as a non-invasive method for assessing global and regional myocardial function before and after surgical intervention. Our preoperative analysis revealed compromised contraction in specific areas of the left ventricle (LV), in the regions that were supplied by both the left coronary artery (LCA) and the right coronary artery (RCA). Interestingly, despite an increase in ejection fraction (EF) measured by conventional echocardiography, the postoperative speckle-tracking analysis revealed persistent impairment in the anterior territories supplied by LCA, highlighting the potential of this technique in identifying myocardial abnormalities during postoperative follow-up. In conclusion, speckle-tracking echocardiography may be a valuable tool for identifying subtle myocardial changes in ALCAPA patients with a higher sensitivity in detecting regional left ventricular (LV) dysfunction compared to conventional echocardiography.

## 1. Introduction

Anomalous origin of the left coronary artery from the pulmonary artery (ALCAPA) is a rare disease caused by the abnormal development of the left coronary artery (LCA). It typically manifests in infancy with heart failure (HF), myocardial ischemia and dilated cardiomyopathy (DCM) [1]. In most cases, the left ventricle (LV) function improves following corrective surgery. However, postoperative data suggest that some myocardial ischemia and fibrosis may persist [2,3]. 

Several imagistic techniques are being used to detect myocardial abnormalities. However, in recent years, advanced echocardiographic techniques such as speckle tracking have become more popular for assessing the myocardium even in the intensive care unit [4,5]. Because of its high sensitivity and low inter-observer variability compared to conventional echocardiography, this method can detect early myocardium abnormalities based on coronary artery vascularization territories, despite a normal ejection fraction (EF) calculated with the standard echocardiography methods. In this case, we aim to present global and regional myocardial function changes in the case of ALCAPA in an infant patient using speckle-tracking echocardiography.

## 2. Case Presentation

We present the case of a two-month-old infant admitted to our intensive care unit with severe HF.

**Figure 1:**
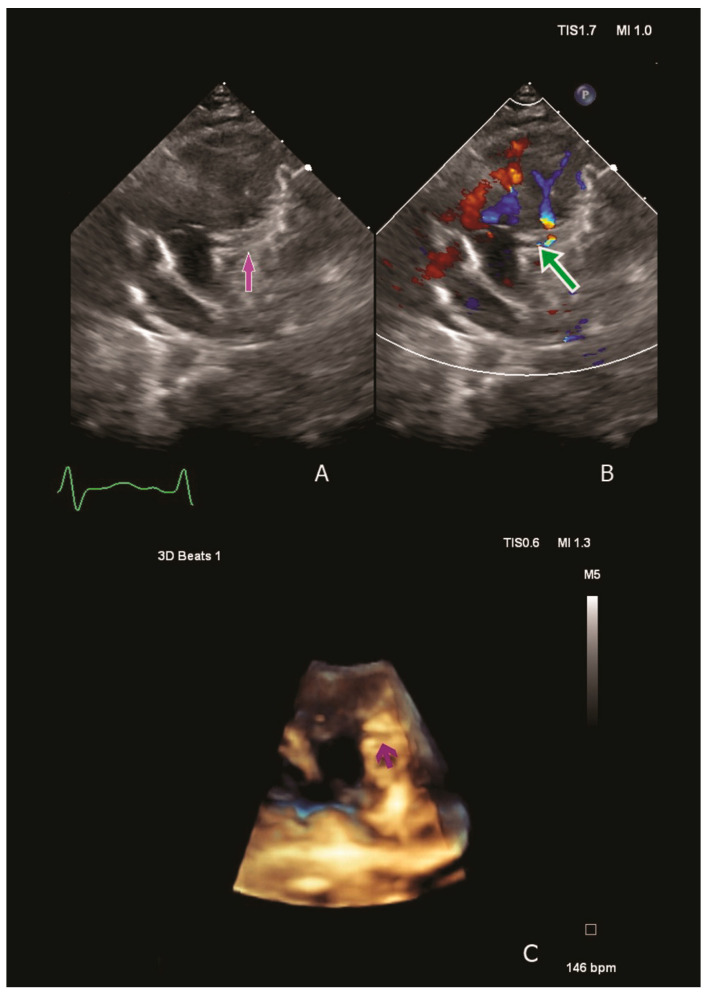
Two/three-dimensional echocardiography confirming ALCAPA diagnosis. We visualise the origin of the left coronary artery from the pulmonary artery: the purple arrow (**A**,**C**) and colour Doppler flow confirming the retrograde flow from the pulmonary artery into left coronary artery- the green arrow (**B**).

Physical examination revealed low cardiac output and acute HF symptoms (pale skin with cold extremities, respiratory distress, tachycardia, a 4/6 holosystolic murmur at the left side of the sternal border, hepatomegaly). Laboratory tests indicated elevated serum creatinine levels, suggesting an acute pre-renal failure due to severely reduced systemic output. Moreover, significantly elevated NT-proBNP, Troponin T hs and Lactate levels were observed (60,477 pg/mL, 138 pg/mL and 6.3 mmol/L). Chest radiography revealed cardiomegaly, and the electrocardiogram revealed septal ischemia with a characteristic ALCAPA QR pattern in lateral leads. A transthoracic echocardiography revealed severe dilatation of the LV compressing the right cavities and severe mitral valve regurgitation with a dilated mitral annulus. The origin of the LCA from the pulmonary artery was visualised using two-dimensional and three-dimensional techniques. Color Doppler revealed retrograde flow from the pulmonary artery into the LCA (Figure 1). 

**Figure 2:**
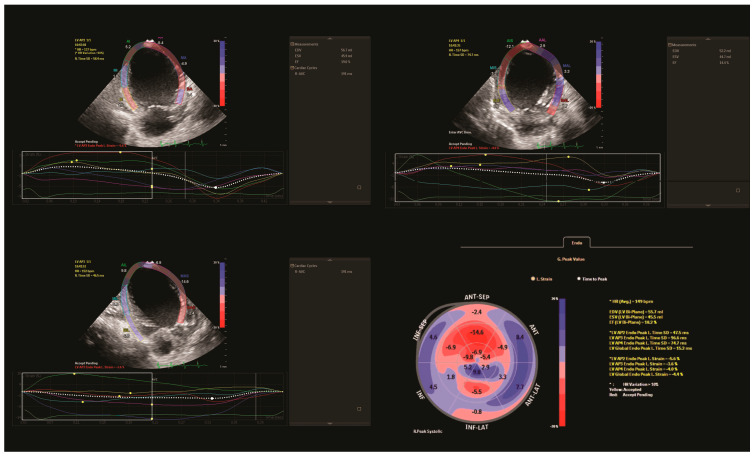
Preoperative speckle-tracking analysis. LV longitudinal strain from the apical two, three and four-chamber view. Time-strain curves show a pathological deformation in the anterior, anterolateral, and inferior LV regions with a hypokinetic septum. Final bull’s-eye plot reveals low global longitudinal strain with severe impairment of both left and right coronary artery territories that supply the left ventricle. AP2: apical two-chamber view; AP3: apical three-chamber view; AP4: apical four-chamber view; EDV (bi-plane): end-diastolic volume (bi-plane); ESV (bi-plane): end-systolic volume (bi-plane); EF (bi-plane): ejection fraction (bi-plane); Global Endo Peak L. Strain (GLS): global longitudinal strain; and HR: heart rate.

Using the Simpson method, LV EF was calculated using conventional echocardiography (18.3%). The speckle-tracking analysis assessed the regional myocardial function of individual segments and global longitudinal strain (GLS). Longitudinal strain measurements of the LV were low in all views (two-chamber: −5.6%, three-chamber: −3.6% and four-chamber: −4.6%), with impaired GLS (−4.4%). The analysis revealed hypokinetic anterior, anterolateral, and inferior LV regions with a hypokinetic septum, corresponding to territories supplied by both the LCA and the right coronary artery (RCA) (Figure 2).

Due to the critical state of the patient, preoperative inotropic treatment with Levosimendan was initiated along with anticoagulation with Enoxaparine. After stabilizing the patient, the surgical intervention was performed three days after the presentation. The LCA was re-implanted into the aorta using an aortic flap and a Proxicor patch as an extension of LCA, with further reconstruction of the pulmonary artery with a pericardial patch.

In the postoperative period, the patient was hospitalized in the ICU for 63 days. In the first three days, sternum closure was delayed to stabilise the patient. Also, during the immediate postoperative care, the patient required aggressive treatment for HF, including the administration of various inotropes such as Adrenaline, Milrinone and Levosimendan. Considering the compromised global contractility with persistent LV dilation and dyskinetic interventricular septum, treatment with Adrenaline was continued and tapered off on day 70 postoperatively. In the first 20 days after surgery, laboratory tests revealed a significant decrease in NT-proBNP values (15,000 pg/mL). Furthermore, transthoracic echocardiography confirmed the patency of the new LCA button, a slightly increased LV EF (25%) and GLS (−10). Additionally, there was an improvement in mitral valve insufficiency from severe to moderate. Compared to the preoperative period, the electrocardiogram still showed a QR pattern in lateral leads, albeit with a reduced q wave amplitude. However, infectious complications such as sepsis with Klebsiella BLSE led to a slight reduction in GLS (−5) and LV EF (20%). Also, in the early postoperative period, the patient developed thrombosis in the left atrium, likely due to decreased LV function. Consequently, a continuous infusion of Heparin was administered, followed by Enoxaparin, resulting in complete thrombus resolution. Additionally, pleural and pericardial effusion necessitated pleural drainage and increased diuretic therapy. In evolution, after the tapering of Adrenaline, a combination therapy for HF comprising a Beta-blocker, ACE inhibitor and aldosterone antagonist was gradually introduced. When the patient was discharged, laboratory tests revealed a significantly lower NT-proBNP value (7226.4 pg/mL) and the speckle-tracking analysis indicated improvement in regional and GLS (−7.9%) compared with the initial measurements, with an LV EF of 30% (Table 1).

**Figure 3:**
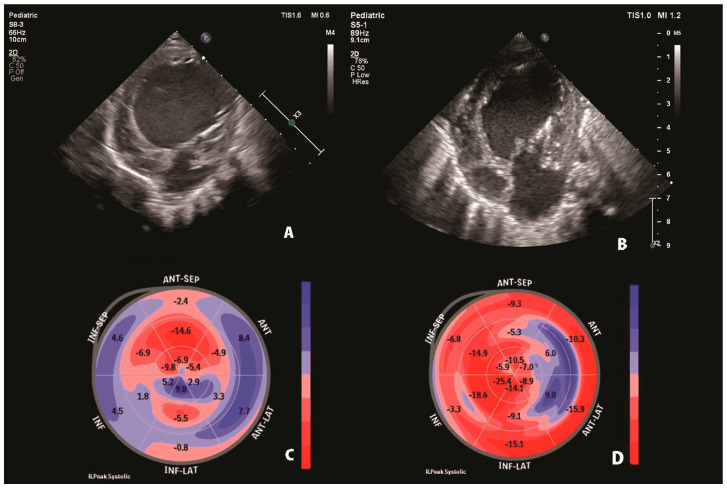
Compared to preoperative echocardiography, follow-up 2D echocardiography showed a mildly dilated left ventricle, with reverse remodelling after surgery (**A**,**B**). Follow-up speckle tracking analysis revealed that postoperative global longitudinal strain increased compared to preoperative analysis, with normal global longitudinal strain in right coronary artery territories. At the same time, mechanical dyssynchrony remained in some segments supplied by the left coronary artery (**C**,**D**).

However, some of the LCA-supplied segments remained impaired (Figure 3).

## 3. Discussion

Anomalous origin of the left coronary artery from the pulmonary artery is a rare and life-threatening congenital heart defect that leads to DCM and high mortality in patients below one year if surgery is not performed [3]. Neonates with ALCAPA are often asymptomatic. However, after the pulmonary pressures drop, retrograde flow from LCA into the pulmonary artery increases, leading to ischemia in the segments supplied by LCA and subsequent cardiogenic shock [2]. In critically ill infants, diagnosing DCM caused by ALCAPA can present challenges due to symptoms overlapping with other cardiac conditions [6]. While conventional echocardiography is used for diagnosis, regarding the real-time information for monitoring and guiding the management, newer techniques like speckle-tracking echocardiography are gaining traction [4]. 

However, the application of strain analysis in the paediatric population remains limited. The initial use of speckle-tracking echocardiography in paediatric patients was described in cases of acute HF with septic shock [5]. In the context of intensive care unit patients with HF, strain measurements are significant as they demonstrate increased sensitivity to changes in cardiac loading conditions, surpassing conventional echocardiography in this context. In our case, both the pre-operative ejection fraction and GLS were significantly low, indicating impaired longitudinal strain in the regions supplied by the LCA and RCA. This finding could indicate that chronic hypoperfusion had a transmural effect on the entire myocardium wall.

After the surgical repair of ALCAPA, the majority of patients recover in the first year following surgery, with improved LV function. There are some hypotheses regarding the dramatic recovery of LV. On the one hand, cardiomyocyte proliferation can contribute to myocardium regeneration. On the other hand, myocardial tissue can reduce metabolic demands in chronic ischemia, the so-called hibernation state; therefore, hibernating myocardium can recover once blood flow is restored [7,8]. Collagen tissue, on the other hand, may persist despite corrective surgery, resulting in fibrosis and impaired microcirculation blood flow. These findings suggest that, despite improved blood flow, the myocardium does not fully recover. Standard echocardiography is useful in assessing the patient’s cardiac function and hemodynamic status [9]. In a large retrospective study, Radman et al. analysed one hundred seventy-seven infants that underwent ALCAPA surgery repair, and stated that, in most patients, LV function measured with standard echocardiography, recovered three years after discharge [10]. However, conventional echocardiography may not be sensitive enough to detect subtle changes in cardiac function postoperatively. 

Therefore, to detect myocardial abnormalities, other imagistic techniques were used such as cardiac scintigraphy or cardiac magnetic resonance [11,12]. However, these methods are challenging to perform in critical infants due to their time-consuming nature and the need for anaesthesia for optimal imaging acquisition. Also, acquiring all the necessary images for speckle-tracking echocardiography can be time-consuming. However, for critically ill patients, efforts can be made to ensure a rapid yet thorough evaluation, balancing the need for detailed imaging with the patient’s stability. Therefore, in our case, we employed speckle tracking in the postoperative period to identify residual coronary disease post-surgery and to detect early subclinical ischemia. We assessed GLS in relation to EF, and both parameters improved simultaneously. Notably, postoperative GLS was normal in the RCA territories but remained impaired in the LCA anterior territories. This persistent impairment of the regional strain in LCA territories suggests a persistent degree of ischemia or fibrosis, despite the improvement of the LV EF measured using standard echocardiography. This finding follows other studies. Castaldi et al. reported that fibrosis persists in the LCA territory after revascularization, and Beyhoff et al. linked subendocardial fibrosis to the decreased longitudinal strain, while transmural fibrosis was associated with decreased GLS [3,13]. Dabrovska et al. found persistent abnormal GLS in LCA territories in paediatric ALCAPA patients after surgery and Shukla et al. noted that LV function did not show significant improvement in the early postoperative period, suggesting incomplete myocardial recovery despite improved blood flow [2,8]. 

Therefore, the evaluation of the LV strain may distinguish treatment responsiveness among different clinical phenotypes. Still, the primary limitation of this study lies in the single case study approach which may limit the generalizability of the findings to a broader population of ALCAPA patients. Additionally, a more thorough understanding of treatment efficacy and the persistence of myocardial issues would be gained through long-term patient follow-up. Integrating speckle-tracking echocardiography in both pre- and postoperative assessments of ALCAPA patients can aid in identifying those at a higher risk of residual coronary disease, facilitating timely interventions and enhancing long-term outcomes, even when traditional echocardiography metrics suggest no abnormalities. Further research is needed to validate these findings. Also, even though LV is more often affected in ALCAPA patients, investigating the potential RV involvement using RV speckle tracking may provide a more complete knowledge of the condition and its impact. However, the speckle tracking of the RV is a novel technique with limited research in paediatric populations [14]. 

## 4. Conclusions

In conclusion, despite clinical improvement and increased ejection fraction following surgical repair in ALCAPA patients, a short-term follow-up revealed that some regions of LV remained dysfunctional. This case highlights the importance of speckle-tracking echocardiography as a non-invasive tool for detecting myocardial abnormalities in both pre- and postoperative follow-up in ALCAPA patients. 

## Figures and Tables

**Table 1:** The most important laboratory measurements and echocardiographic parameters assessed throughout the progression of this case.

	Admission	Postoperative (Day 15 Postop)	Infection Time (Day 23 Postop)	Discharge (Day 70 Postop)
LV EF (%)	18	25	20	30.8
LVEDD (mm)	44 (z score = 7.29)	41	40	35 (z score = 5)
LVOT VTI (m/s)	1.1	0.9	0.8	1
GLS	−4	−8	−5	−7.9
NT-proBNP (pg/mL)	60,477	15,000	-	7226.4
Troponin T (pg/mL)	138	-	-	23

LV EF: Left ventricle ejection fraction; LVEDD: left ventricle end-diastolic dimension; LVOT VTI: left ventricle outflow tract velocity time integral; GLS: global longitudinal strain; NT-proBNP: N-terminal pro-b-type natriuretic peptide; Postop: postoperative period.

## Data Availability

No new data were created.
